# Three-Dimensional Imaging of Dislocations in a Ti–35mass%Nb Alloy by Electron Tomography

**DOI:** 10.3390/ma8041924

**Published:** 2015-04-21

**Authors:** Kazuhisa Sato, Satoshi Semboshi, Toyohiko J. Konno

**Affiliations:** Institute for Materials Research, Tohoku University, 2-1-1 Katahira, Aoba-ku, Sendai 980-8577, Japan; E-Mails: semboshi@imr.tohoku.ac.jp (S.S.); tjkonno@imr.tohoku.ac.jp (T.J.K.)

**Keywords:** electron tomography, dislocations, 3D configurations, Ti–Nb, scanning transmission electron microscopy (STEM)

## Abstract

We have studied three-dimensional (3D) configurations of dislocations in the β phase of a Ti–35mass%Nb alloy by means of single-axis tilt tomography using bright-field scanning transmission electron microscopy (BF-STEM). To visualize dislocations, the *hh*0 systematic reflections were excited throughout tilt-series acquisition with the maximum tilt angle of 70°. Dislocations in the β grains were clearly reconstructed by the weighted back-projection algorithm. The slip planes of the dislocations were deduced by rotating the reconstructed volumes with the aid of selected area electron diffraction patterns. It was found that BF-STEM images with relatively low contrasts, taken along low-order zone axes, are capable to reproduce and preserve the quality of reconstructed image of dislocations. We also found that tilt angles as low as 40° are practically acceptable to visualize 3D configurations of dislocations, while there exists limitation in resolution due to the existence of a large missing wedge.

## 1. Introduction

Electron tomography is a technique to visualize three-dimensional (3D) structures inside materials in a transmission electron microscope (TEM) with the aid of computational reconstruction processes. This technique has been applied for various fields of materials science so far [1]. One of the recent challenges in 3D tomography is the visualization of dislocations in metals and other materials. 3D tomographic observation of dislocations was firstly reported for a GaN single crystal specimen using the weak-beam dark-field (DF) transmission electron microscopy (TEM) imaging method [2,3]. Because of the invisible criterion (***g***•***b*** = 0; ***g*** and ***b*** are, respectively, the excited reciprocal lattice vector and Burgers vector in concern) [4], excitation of a selected ***g***_hkl_ throughout the tilt-series acquisition is mandatory for dislocation tomography. After the pioneering work by Sharp *et al.* 3D visualization of lattice defects including dislocations has been demonstrated by several researchers for materials including Si [5], stainless steel [6,7], Al [8], and nanoparticles [9,10]. These works have mainly focused on the development and verification of novel techniques; therefore, materials for 3D observation have been mostly limited in specimens with a single-crystal or large grains. Our attempt here is to visualize dislocations in a rather narrow area in two-phase alloys. For this purpose, we selected a quenched Ti–35mass%Nb alloy composed of a so-called α'' martensite with an orthorhombic structure and a small amount of the β phase with a body centered cubic (bcc) structure [11]. The alloy system has been attracted for biomedical applications due to their excellent biocompatibility, high corrosion resistance, low Young’s modulus, high strength, toughness, and so on [12]. In general, the β phase contributes primarily to ductility and workability of the two-phase alloy, because it is softer than the α'' martensite. It is also noted that there is few report on the dislocation tomography of bcc crystals. Therefore, a proper characterization of dislocations in the β phase is necessary for better understanding mechanical properties of two-phase Ti-alloys.

In this study, we have examined 3D visualization of dislocations in β phase of a Ti–Nb alloy using single-axis tilt tomography. We employed bright-field scanning transmission electron microscopy (BF-STEM) for imaging dislocations by exciting *hh*0 systematic reflections parallel to the tilt axis. Effects of lowness in contrasts and the maximum tilt angle on the shapes and width of reconstructed dislocations are discussed.

## 2. Experimental Procedure

Ti–35mass%Nb alloys were prepared by arc-melting in an argon atmosphere using high purity titanium (99.99%) and niobium (99.9%). The ingots obtained were annealed at 1323 K for 86.4 ks in vacuum for homogenization followed by cold rolling. The homogenized specimens were solution-treated at 1223 K (above the β transus) for 10.8 ks in an evacuated quartz tube, and then quenched into ice water. Details of the alloy specimen preparation can be found in the literature [11]. To prepare samples for TEM observation, disk-shaped specimens were ground to less than 100 μm thickness, and electropolished in a solution of 5% HNO_3_ in methanol at 243 K with an applied potential of less than 10 V, followed by a low angle argon ion milling.

Microstructures of the alloy including dislocations were characterized by a 300 kV-TEM (FEI TITAN80-300, FEI Company, Hillsboro, OR, USA) with STEM mode. Tilt-series of BF-STEM images were obtained using the Xplore3D software (FEI Company) taking dynamic focus into consideration. A triple-axes holder (Mel-Build model HATA-8075, Mel-Build Corporation, Fukuoka, Japan) [6] was used for the tilt-series acquisition with the maximum tilt angle of 70°. The diffraction vector was maintained close to ***g***_hkl_ = 110 by exciting *hh*0 systematic reflections parallel to the tilt axis during the acquisition of BF-STEM images. The beam convergence was set to be 14 mrad in semi-angle and outer collection angle on the BF-detector to be 11 mrad. We used an FEI Inspect3D software package for subsequent data processing, including alignment of the tilt axis for the obtained data set based on an iterative cross-correlation technique, and 3D reconstruction based on a weighted back-projection (WBP) method. Kinematical convergent-beam electron diffraction (CBED) patterns were calculated using the WebEMAPS software [13].

## 3. Results

**Figure 1:**
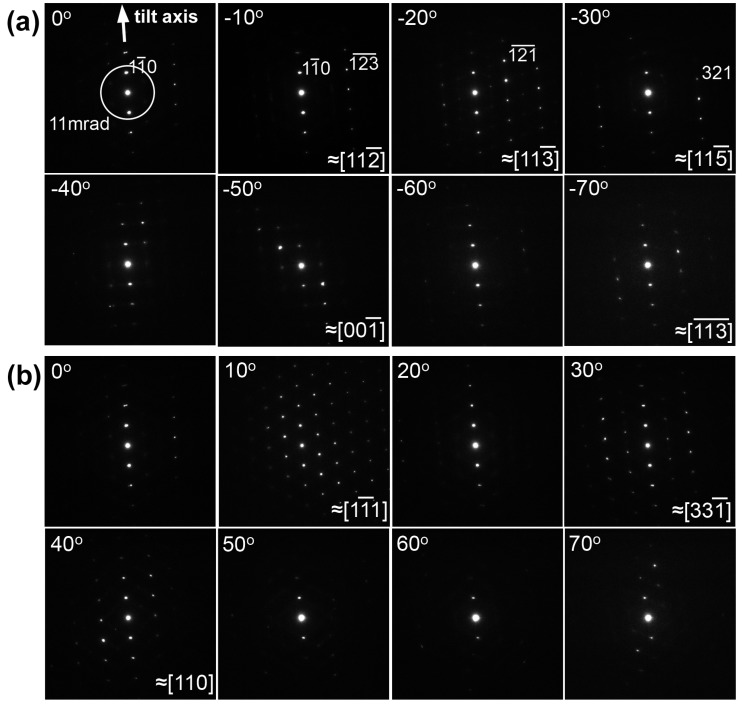
Tilt-series of selected area electron diffraction (SAED) patterns obtained from the β phase region in a Ti–35mass%Nb alloy. The *hh*0 systematic reflections are excited parallel to the tilt axis. (**a**) SAED patterns obtained at tilt angles from 0° to −70°, and (**b**) those from 0° to 70°.

Figure 1 shows a series of selected area electron diffraction (SAED) patterns obtained from the β phase region. All these patterns were obtained using parallel beam illumination. Tilt angles are shown in the upper left corner of each pattern. As seen, *hh*0 systematic reflections are excited along the tilt axis of the triple-axes holder. The circle indicates the outer collection angle of 11 mrad of the BF-detector used for STEM imaging; hence only three beams, 000, 1$\overset{―}{1}$0 and $\overset{―}{1}$10 are contributed to the image formation.

**Figure 2:**
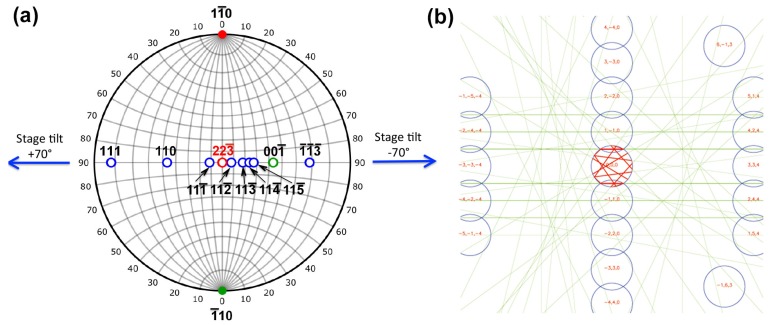
(**a**) Stereographic projection indicating changes of zone axes as a function of stage tilt angle. The initial beam incidence is close to [22$\overset{―}{3}$]_β_, and both 1$\overset{―}{1}$0 and $\overset{―}{1}$10 reflections are always excited during the tilt series acquisition; (**b**) A simulated convergent-beam electron diffraction (CBED) pattern with the [22$\overset{―}{3}$]_β_ zone.

As can be seen, zone axis patterns, such as [11$\overset{―}{2}$], [11$\overset{―}{3}$], [11$\overset{―}{5}$], or [001], appear during the specimen tilting of up to ±70°. It should be noted that ***g***_hkl_ = 1$\overset{―}{1}$0 is satisfied even at a tilt angle as high as 70°. Thus, the invisible criterion (***g***•***b*** = 0) is maintained throughout the tilt-series acquisition. It can also be noted that zone axes of [11$\overset{―}{3}$] and [$\overset{―}{1}$$\overset{―}{1}$$\overset{―}{3}$] are apart from each other by 50.5° in a cubic crystal, and accordingly that these two patterns were obtained at the stage tilt angle of −20° and −70°, respectively, further demonstrating that the tilting axis is parallel to the *hh*0 systematic reflections. Changes of zone axes as a function of stage tilt angle can be summarized on a stereographic projection shown in Figure 2a. As seen, the initial beam incidence is close to [22$\overset{―}{3}$]_β_, and both 1$\overset{―}{1}$0 and $\overset{―}{1}$10 reflections are always excited during the specimen tilt. A simulated CBED pattern with the [22$\overset{―}{3}$]_β_ zone, which corresponds to the BF-STEM imaging condition, is shown in Figure 2b. As seen, the *hh*0 systematic reflections are excited and the non-systematic reflections are far from the systematic row.

**Figure 3:**
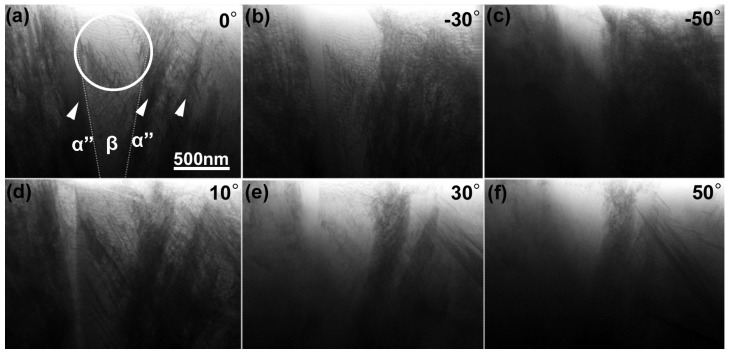
Snapshots of bright-field scanning transmission electron microscopy (BF-STEM) images of the as-quenched Ti–35mass%Nb alloy acquired during a tilt-series observation. SAED patterns were observed from a circular area indicated in (**a**). Arrowheads indicate acicular α'' martensite plates. Dotted lines indicate α''/β grain boundaries. Tilt angles for each image is as follows: (**a**) 0°, (**b**) −30°, (**c**) −50°, (**d**) 10°, (**e**) 30°, (**f**) 50°.

Figure 3 shows snapshots of BF-STEM images of the as-quenched Ti–35mass%Nb alloy acquired during a tilt-series observation. They are shown after the alignment of tilt axis, which is set vertical here. The tilt angles are (a) 0°, (b) −30°, (c) −50°, (d) 10°, (e) 30°, and (f) 50°. This tilt-series was obtained sequentially from 0 to −70° and then 0 to +70° with an angular increment of 2°. The SAED patterns shown in Figure 1 were obtained from an area indicated by the circle in Figure 3a (~750 nm in diameter) near the specimen edge. Arrowheads in the image shown in Figure 3a indicate dark contrast arising from acicular α'' martensite, which can be typically observed in a quenched Ti–Nb alloy [11]. Thus, it can be stated that the encircled area composed of the β phase exists in between the α'' martensite plates. Dotted lines indicate α''/β grain boundaries. Note that α'' martensite is the primary phase of this quenched alloy. Morphology and distribution of acicular α'' martensite can be found in the literature [11].

**Figure 4:**
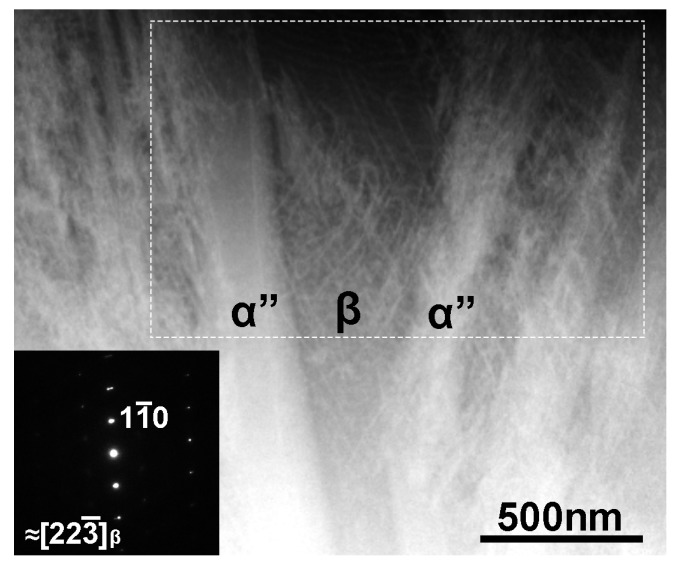
Magnified BF-STEM image and corresponding SAED pattern of the Ti–35mass%Nb alloy observed at a stage tilt of 0°. The image contrast is reversed in order to clearly show fine dislocations. The beam incidence is close to the [22$\overset{―}{3}$]_β_. The rectangular area indicated by dashed lines was selected for subsequent 3D reconstruction.

Figure 4 shows a magnified BF-STEM image and a corresponding SAED pattern of the Ti–35mass%Nb alloy observed at a stage tilt of 0°. The image contrast shown here is reversed in order to clearly show fine dislocation arrangement. The beam incidence is close to the [22$\overset{―}{3}$]_β_. As seen, there are dense dislocations in the β phase region. The rectangular area indicated by dashed lines was selected for subsequent 3D reconstruction.

**Figure 5:**
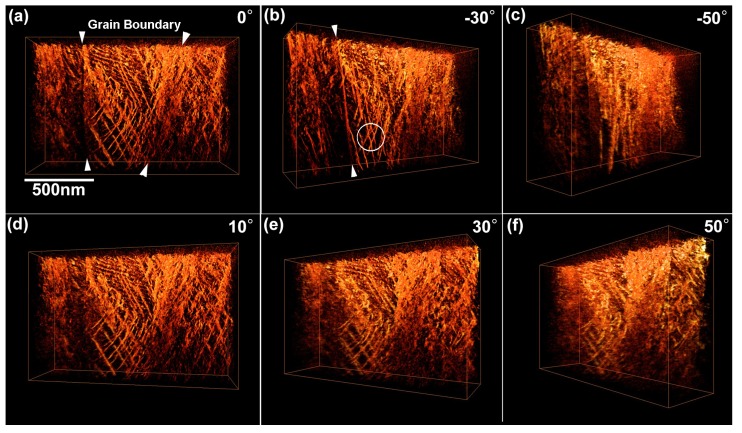
Three-dimensional views of the dislocations reconstructed from a BF-STEM tilt-series dataset using a weighted back-projection (WBP) technique. Arrowheads indicate the α''/β grain boundary. A kink on a ($\overset{—}{1}$01) slip plane is seen in the encircled area. Tilt angles for each image is as follows: (**a**) 0°, (**b**) −30°, (**c**) −50°, (**d**) 10°, (**e**) 30°, (**f**) 50°.

Figure 5 shows reconstructed 3D images of dislocations in the β phase region. Tilt angles indicated for each image correspond to those shown in Figure 3. The reconstructed volume size is 1003 nm × 1532 nm × 489 nm (377 × 576 × 183 pixels, *i.e.*, 2.66 nm/pixel). The reconstruction have revealed that the reconstructed region of the specimen had a wedge shape with thickness increasing from approximately 100 to 200 nm (top to bottom in the image). It should be mentioned that at the tilt angle of 70° the specimen thickness reaches approximately 290 to 580 nm; the thickness is within the maximum observable limit for electrons accelerated at 300 kV (~1 μm for Fe) [14]. The arrowheads shown in Figure 5a indicates grain boundary between α'' and β phases, which can be seen in the original image shown in Figure 4. Dislocations with different directions overlap and some of them are bent. Rather wavy shape implies that they are mostly mixed dislocations. The dislocation density estimated was rather high value of ~10^14^ m^−2^. According to the literature, dislocation densities of 10^12^ m^−2^ and 10^15^ m^−2^ were reported for the β phase in Ti–10V–2Fe–3Al alloys before and after deformation, respectively [15]. This implies that the dislocations in the β phase observed in this study are introduced by compressive stress arising from the volume changes of adjacent crystals due to the martensitic transformation during quenching.

**Figure 6:**
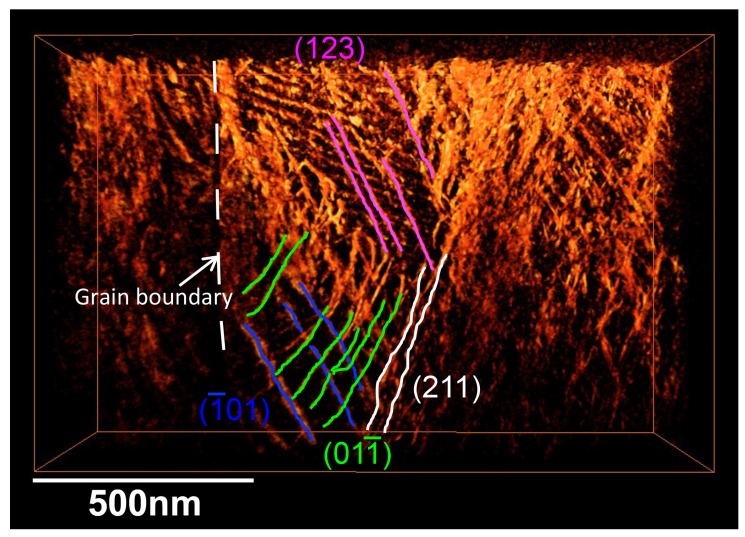
A result of slip plane analyses: dislocations on different slip planes are marked by different colors.

By rotating the reconstructed volume, we can analyze slip planes of these dislocations with the aid of corresponding SAED patterns. When a trace of dislocations viewed from an edge-on direction is orthogonal to a diffraction vector ***g***_hkl_, the slip plane can be assigned as (*hkl*). For example, the slip plane analysis led us to conclude that the bending of dislocations as seen in Figure 5b (encircled area) is a kink of dislocations on a ($\overset{—}{1}$01) slip plane. The existence of the kink is practically difficult to detect on the original STEM images and this is a benefit of using tomographic reconstruction. The slip planes determined are ($\overset{—}{1}$01), (01$\overset{—}{1}$), (211), and (123), and dislocations exist on these planes are marked by different colors in Figure 6. It can be pointed out that these are typical slip planes for metals with the bcc structure.

## 4. Discussion

**Figure 7:**
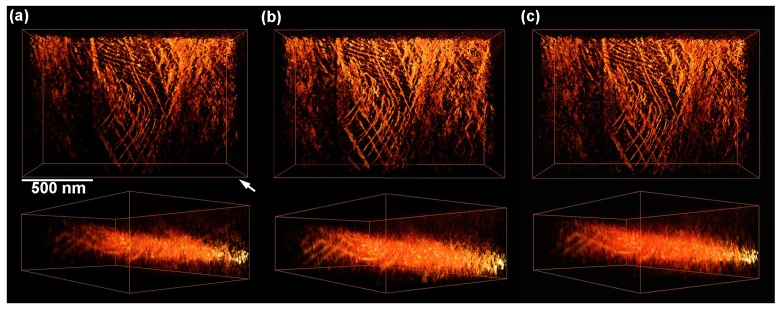
Effects of low contrast images and the maximum tilt angles on shapes and width of reconstructed dislocations. The maximum tilt angles and the number of images used for the reconstruction are as follows: (**a**) 70°, 63 images, (**b**) 70°, 71 images, (**c**) 40°, 41 images. An arrow indicates the viewing direction of the attached cross section images.

Here, we discuss practical aspects of 3D dislocation imaging from the viewpoints of contrast quality of data set and of the extent of tilting angle. In the tilt-series obtained in this study, SAED patterns with zone axes appeared at −10° ([11$\overset{—}{2}$]), −20° ([11$\overset{—}{3}$]), −30° ([11$\overset{—}{5}$]), −50° ([00$\overset{—}{1}$]), −70° ([$\overset{———}{113}$]), −10° ([1$\overset{—}{1}$1]), 30° ([33$\overset{—}{1}$]), and 40° ([110]) as shown in Figure 1. When the tilt angle reaches an angle satisfying a low-order zone axis (satisfying the Bragg conditions for *hkl* reflections other than the *hh*0 systematic reflections), background intensity was enhanced in the corresponding STEM images, and hence the contrasts of dislocations were reduced. Such low contrast images can be discarded in the reconstruction process as recently reported by Kacher *et al.* [7]. To check the effect of such Bragg reflections other than the *hh*0 systematic reflections on the quality of reconstructed dislocation images, we examined 3D reconstruction removing images obtained under low-order zone axes incidence. The result is shown in Figure 7a. An arrow indicates the viewing direction of the attached cross section images. For comparison, the result, reconstructed using all the experimental images, is shown in Figure 7b, which is the same image shown in Figure 5a. As can be seen, the effects of low contrast images taken at zone axes on shapes and width of dislocations are negligibly small within the resolution of the present study. This is probably due to the nature of the BF-STEM imaging in the present study with the outer collection angle of 11 mrad. This condition excludes most of the Bragg reflections other than 000, 1$\overset{—}{1}$0, and $\overset{—}{1}$10 throughout the tilt-series (The [1$\overset{—}{1}$1] incidence is the only exception; four kinds of 110 reflections are incorporated within the aperture in addition to 000, 1$\overset{—}{1}$0, and $\overset{—}{1}$10 reflections).

We also examined the effect of maximum tilt angles on the reconstruction. Some of the recent studies on electron tomography of dislocations were carried out to visualize dislocations with tilt angles less than 40° [7,16]. In general such a limitation of the maximum tilt angle degrades the resolution of reconstructed images in the *z* direction (parallel to the optic axis) due to the effect of missing wedges as reported in the preceding studies using a phantom [1] or a real 3D object [17]. Figure 7c shows the results of a trial reconstruction using only 41 images with the maximum tilt angle of 40°. The shapes and width of dislocations have been well reconstructed. As seen, there is no apparent difference in the reconstructed images shown in Figure 7b (70°) and 7c (40°). In the latter case, dislocation images in the *z*-direction parallel to optic axis became blur by at most 30% due to the missing wedge, while the overall features still remain in the reconstructed volume. Thus it can be stated that the maximum tilt angle as low as 40° is practically acceptable to qualitatively visualize 3D configurations of dislocations without losing essential information, such as slip planes; while, ideally speaking, higher tilt angles are required for better resolution.

## 5. Conclusions

We have studied 3D configurations of dislocations in the β phase of a Ti–35mass%Nb alloy by means of single-axis tilt tomography using BF-STEM. The results can be summarized as follows:

(1) Dislocations in a β grain were imaged by exciting *hh*0 systematic reflections to maintain an invisible criterion (***g***•***b*** = 0) throughout the tilt-series acquisition with the maximum tilt angle of 70°.
(2) Dislocations were clearly reconstructed by weighted back projection algorithm. The slip planes determined were ($\overset{—}{1}$01), (01$\overset{—}{1}$), (211), and (123). A kink on a ($\overset{—}{1}$01) slip plane was found in the 3D reconstructed volume.
(3) Effect of low contrast images taken at low-order zone axes on reconstructed dislocation images is small within the resolution of the present study. This is probably due to the BF-STEM imaging condition which excludes most of the Bragg reflections other than 000, 1$\overset{—}{1}$0, and $\overset{—}{1}$10 throughout the tilt-series acquisition.
(4) Low tilt angles of around 40° are practically acceptable to visualize 3D configurations of dislocations, in spite of the loss of resolution in the *z* direction due to the existence of a large missing wedge.

## Supplementary materials

Supplementary File 1

Supplementary File 2
